# Study on the Shielding Effectiveness of an Arc Thermal Metal Spraying Method against an Electromagnetic Pulse

**DOI:** 10.3390/ma10101155

**Published:** 2017-10-04

**Authors:** Han-Seung Lee, Hong-Bok Choe, In-Young Baek, Jitendra Kumar Singh, Mohamed A. Ismail

**Affiliations:** 1 Department of Architectural Engineering, Hanyang University, 1271 Sa 3-dong, Sangrok-gu, Ansan 426-791, Korea; ercleehs@hanyang.ac.kr (H.-S.L.); hongbokchoe@gmail.com (H.-B.C.); biy74@naver.com (I.-Y.B.), jk200386@hanyang.ac.kr (J.K.S.); 2 Department of Civil and Construction Engineering, Faculty of Engineering and Science, Curtin University Malaysia, Miri 98009, Malaysia

**Keywords:** electromagnetic pulse (EMP), shielding effectiveness (SE), arc thermal metal spraying method, EMP shielding coating thickness

## Abstract

An electromagnetic pulse (EMP) explodes in real-time and causes critical damage within a short period to not only electric devices, but also to national infrastructures. In terms of EMP shielding rooms, metal plate has been used due to its excellent shielding effectiveness (SE). However, it has difficulties in manufacturing, as the fabrication of welded parts of metal plates and the cost of construction are non-economical. The objective of this study is to examine the applicability of the arc thermal metal spraying (ATMS) method as a new EMP shielding method to replace metal plate. The experimental parameters, metal types (Cu, Zn-Al), and coating thickness (100–700 μm) used for the ATMS method were considered. As an experiment, a SE test against an EMP in the range of 10^3^ to 10^10^ Hz was conducted. Results showed that the ATMS coating with Zn-Al had similar shielding performance in comparison with metal plate. In conclusion, the ATMS method is judged to have a high possibility of actual application as a new EMP shielding material.

## 1. Introduction

An electromagnetic pulse (EMP), generated by a nuclear explosion, can neutralize electronic and communication devices instantly within a wide radius, approximately up to 1500 km. When the EMP arrives at the ground surface, it creates a very strong induced current, which enters electronic circuits and destroys them by means of overcurrent that the circuit cannot resist [1,2]. It is such as a high-output energy bomb, which consists of an electromagnetic wave (=electronic wave) and explodes at the range of 40–400 km altitudes [1,2]. In addition, it is able to produce 50–100 kV/m of electric field intensity at its maximum. Considering that the receiving electric field intensity of a general radio transceiver is just several mV, it can be seen that EMP has a very strong amount of electromagnetic energy [2]. In addition, the frequency range of an EMP is from about 100 kHz–100 MHz. This means that an EMP can influence and damage the operational frequency ranges of civilian and military electronic equipment [3].

From the point of military defense, an EMP can cause neutralization and risk of national and international infrastructure facilities, such as the electrical grid and the communication network [4]. Moreover, it can cause severe damage to confidential military intelligence systems by paralyzing military communication equipment [5]. Therefore, it is highly recommended to develop EMP shielding methods to minimize the damage.

The EMP, or electromagnetic interference (EMI), can be reduced using different conducting, flexible, ductile, composite, and novel materials [6,7,8,9,10,11]. The SE of these composite materials depend on intrinsic conductivity, aspect ratio, and content of the fillers [12,13]. These properties of materials can be improved by adding conducting elements, such as carbon nanotubes, graphene, etc. [14]. The conducting polymer is a promising material to be used to increase the SE of composite materials in high-frequency ranges [15,16,17]. However, in spite of the advantage of these materials, it cannot be used as SE materials against an EMP due to some disadvantages, such as high cost, impurities from the catalysts, bundling, and aggregation [18].

**Figure 1:**
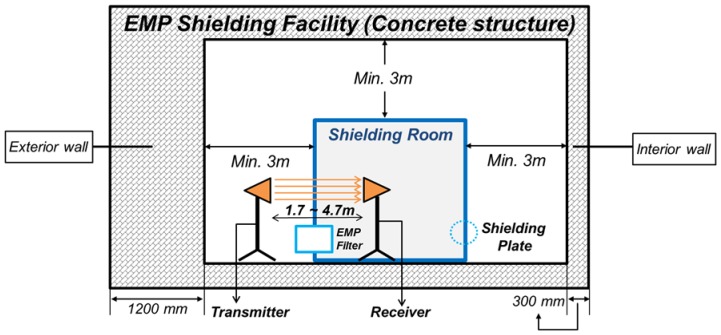
Floor plan of a conventional electromagnetic pulse (EMP) shielding facility.

Conventional EMP shielding facilities for floor systems can be explained through Figure 1. EMP shielding methods are divided largely into two parts. One is a shielding plate at the door, which consists of a shielding room. A shielding room is a place where electronic equipment is installed for their protection. Another is an EMP filter and honeycomb, a method to shield the point of entry (POE) of an EMP in the shielding room. POE is a hole that is inevitably made when communication equipment and heating, ventilation and air conditioning (HVAC) are brought into the shielding room [19].

In previous studies, the main research of EMP shielding methods were with respect to shielding effectiveness (SE) of concrete structures, EMP filters, and honeycomb [20,21,22,23]. Concrete structures are the basic frame of EMP shielding facilities to protect the shielding room from being destroyed by external physical influence. In general, when the concrete structure is constructed, a shielding room in manufactured by metal plates placed on the inside of the concrete structure.

On the other hand, the study on shielding plate has not actively proceeded [24,25]. Conventionally, metal plate has been mainly used as shielding plate due to its excellent SE against an EMP. For the construction of the shielding room, metal plates are welded or assembled by bolts to each other. However, this method has shown a difficulty in manufacturing as follows: a possibility of EMP inflow through welded parts, and expensive construction costs [26]. To solve this problem, developing a new EMP shielding method to replace the metal plate is required.

The objective of this study is to examine an applicability of the arc thermal metal spraying method (ATMS method), as a new EMP shielding method replacing conventional metal plate. The ATMS method has several properties that can perform as a shielding material. For example, this method can shield electromagnetic waves, form a shielding coating easily, and control the coating thickness flexibly. In addition, it can be applied to any substrate under the condition that the substrate surface is treated to have roughness. Thus, there is no need to install a shielding room. The ATMS method can be applied directly to a concrete wall. It is expected to reduce much of the construction cost for EMP-shielded rooms.

The principle of the arc ATMS method is described elsewhere [27,28,29,30,31,32]. However, the ATMS method is an anti-corrosion method that forms a metal coating on a steel structure by using a metal wire mesh, such as Zn and Al. The process is as follows: first, two metal wires are supplied into the driving device, which plays the role of an arc spray gun. Second, through a linear element guide nozzle, those wires are shifted and melted at the arc point. By compressed air, the melted metal particles are sprayed to the outside entraining cooling action and attached to the surface of the substrate. These attached metal particles, by continued accumulation and consolidation, finally form a porous and compact metal coating (arc thermal metal spray coating; hereafter, ATMS coating). During this process, when metal particles contact the surface of the substrate, it is quickly cooled to almost room temperature due to compressed air. Since the ATMS coating is formed at room temperature, the substrate does not generate any thermal strain. Finally, a solid and stable ATMS coating is formed [33,34].

There are two reasons why the ATMS method is expected to show suitable properties as an EMP shielding method. First, because it uses a metallic material the same as metal plate, it would show a SE against EMP as well. Second, because it is a coating method to prevent corrosion of the steel structure, the ATMS coating is resistant to chloride or moist environments. When the coating is exposed for a long period under a corrosive environment, it creates an oxide coating on itself and becomes more compact [35]. In some cases, for the purpose of military defense, EMP shielding facilities are constructed underground. Such an environment is vulnerable to corrosion. Thus, the ATMS method can combine the anti-corrosion effect and the EMP shielding effectiveness together.

The shielding process to reduce the EMP impact on the surface is described in Supplementary Figure S1. This figure indicates the shielding principle of metallic material against electromagnetic waves. In the conventional method, the entire shielding room is constructed with metal plate (galvanized steel, copper, iron, etc.), about 2–5 mm in thickness. This is because the metallic material shows excellent SE against electromagnetic waves.

This attenuates the inflow of the electromagnetic wave by three mechanisms. First, it is the reflection loss, generated by impedance mismatching between air layer and metal. Most of electromagnetic wave is shielded by the reflection loss. Second, it is the penetration loss, generated in the form of heat dispersion caused by the ohmic loss, when the electromagnetic wave penetrates into the metal shielding plate. Finally, it is the multi-reflection loss, generated by the second reflection inside the inner metal layer. This happens at the boundary layer of both sides of the metal shielding plate [36].

Therefore, it can be seen that metallic materials are effective as EMP shielding materials. However, the SE of metallic materials varies depending on the coating conditions or type of metal. In general, a metallic material that shows low impedance with electrical conductivity and a metal with no holes or cracks can improve the SE to prevent inflow of high-frequency electromagnetic waves.

For the purpose of this study, a SE test against an EMP was carried out to compare the shielding performance between the ATMS coating and metal plate.

## 2. Experimental Details

### 2.1. Experimental Variables

Table 1 indicates the experimental variables for the evaluation on the SE against EMP. In this experiment, the type of metal used as the shielding material and the thickness of ATMS coating were selected to be tested. In case of EMP shielding material, Fe and Cu were selected for the metal plate and Cu and Zn-Al were selected for the ATMS method. The mesh of wires chosen for the deposition of the coating was 2.4 mm. In the case of the thickness of the ATMS coating, 100, 300, 500, and 700 μm were selected with consideration of the application efficiency. The coating thickness was measured at three different locations of each coating and error is shown in Figure S2. The error was a maximum of ±5% for each coating thickness.

### 2.2. Test Specimens for Experiments

Table 2 indicates the list of specimens used for the SE test. Specimen 1 is a tempered glass, which is a substrate for ATMS coating. Glass is known as having no SE against electromagnetic waves. For that reason, it was used to measure a SE of the ATMS coating by itself. In addition, because the substrate required enough durability when blasting on the surface is treated, the tempered glass was expected to perform as an appropriate material as a substrate. A thickness of 6 mm was used in this experiment and the surface was sand-blasted to secure adhesiveness between the ATMS coating and the tempered glass. Specimens 2 and 3 are Fe and Cu metal plate, which are widely used as EMP shielding materials. Specimens 4–11 are specimens that were prepared by the ATMS method. ATMS coating by Cu was selected for a comparison of SE with the conventional metal plate by Cu. ATMS coating by Zn-Al was selected because it has been widely used as the spraying metal type in the ATMS method. Thickenesses of 100, 300, 500, and 700 μm of the ATMS coating were selected. When the ATMS method was applied, in the case an ATMS coating over 700 μm thickness is formed at one cycle, there was a limitation of adhesiveness between ATMS coating and tempered glass because of the thermal strain of the ATMS coating itself. For that reason, the maximum thickness was set to 700 μm. Metal plates with a thickness of 3 cm were selected, considered to be commonly used in the construction of the shielding room.

### 2.3. Experimental Procedures

**Figure 2:**
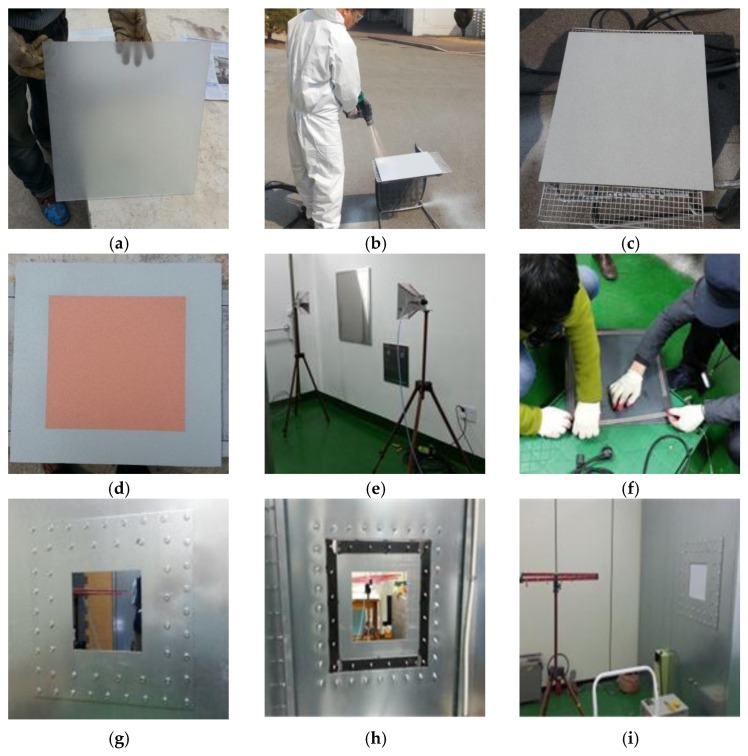
Experimental process. (**a**) Substrate preparation; (**b**) application of ATMS; (**c**) specimen after ATMS (Zn-Al coating); (**d**) specimen after ATMS (Cu coating); (**e**) calibration work; (**f**) gasket installation; (**g**) EMP shielding wall (outside); (**h**) EMP shielding wall (inside); and (**i**) SE test.

Figure 2 indicates the whole process of the experiment. First, the tempered glass was prepared with sand-blasting, as shown in Figure 2a. Second, ATMS coating was applied, as shown in Figure 2b. Figure 2c is the case of applying Zn-Al and Figure 2d is the case of applying Cu (colored in red). Third, calibration of the electromagnetic wave transmitter and receiver was conducted, as shown in Figure 2e. Finally, the gasket installation, EMP shielding wall outside and inside, and SE test is shown in Figure 2f–i, respectively.

### 2.4. Standards on Shielding Effectiveness against EMP and SE Tests

Figure S3 indicates the required SE against EMP of shielding material for each frequency range when constructing the EMP shielding room. The specification in this criteria follows IEEE-STD-299, be the Institute of Electrical Engineers (IEEE); MIL-STD-188-125-1, a common specification of the US Ministry of Defense; and DMFC 4-70-30, a design standard for electromagnetic wave defense facilities that is based on the criteria of national defense and military facilities.

The required minimum SE value ranges from 10^3^ to 1.5 × 10^9^ Hz as shown in Figure S3 [37,38]. In the magnetic area, the frequency range is from 10^3^ to 2 × 10^7^ Hz (=1 kHz to 20 MHz). The required SE increases proportionately as the frequency increases. For the resonant range and plane wave area, the frequency range is from 2 × 10^7^ to 10^9^ Hz (=20 MHz to 1 GHz). In this range, over 80 dB SE is required regardless of the frequency change. The unit of SE is dB (decibel) and this represents the attenuation amount of electromagnetic wave by the shielding material.

Equation (1) indicates the calculation on SE against EMP of the shielding materials in a SE test. This is to confirm the SE of the shielding materials against an emitted electromagnetic wave. It represents the ratio of received amount when there is no shielding material (Vc) to the received amount with a shielding material (Vm) [39].

Shielding Effectiveness (SE) = 20 log (Vc/Vm) (dB)(1)

Figure S4 shows the schematic diagram of the SE test for this experiment. In general, as shown in Figure S4, the SE test is conducted by installing the electromagnetic wave transceiver at the inside and outside of the EMP shielding wall. Prior to the test, the EMP shielding room was manufactured and completely closed without any open space.

In this experiment, for the accurate measurement of specimens, an area of 300 × 300 mm^2^ was cut out from the shielding wall and the specimen was fixed into void part. The reason is to allow the electromagnetic wave would be transmitted only within the specimen area upon emission of the electromagnetic wave. If a size of a void part was smaller than 300 × 300 mm^2^, there would be a possibility that a wave could pass the shielding wall surface. In the case of the specimen size, an area of 450 × 450 mm^2^ was prepared. In addition, the calibration work was conducted by emitting an electromagnetic wave in a state that there would be no specimen on the wall. The purpose was to adjust the transmission and reception error rates in the generator before the SE test.

Figure S5 indicates the cross-sectional diagram of SE test for the installation of specimens. In order to fix the specimen above the shielding wall, a specimen fixation plate was installed at the inside and outside of the shielding wall with bolts. This was to prevent the specimen from moving. After the fixation, the experiment was carried out.

Table S1 indicates the frequency range of transmitted electromagnetic wave in SE test. The test frequency range was selected based on the IEC 61000-4-23 of IEC (International Electronic Committee) regulations, MIL-STD-188-125-1, and IEEE-STD-299 [37,38].

By referring to the required SE as shown in Table S1, specimens 1–3 correspond to the magnetic area (10^3^–2 × 10^7^ Hz), specimens 4 and 5 correspond to the resonant range and plane wave area (2 × 10^7^–10^9^ Hz), and specimens 6 and 7 correspond to the plane wave area (10^9^–1.5 × 10^9^ Hz). Although there were differences in the number of measurements depending on the regulation, in order to secure the reliability of the measurement results, the average value was extracted after three measurements per each frequency range.

## 3. Results and Discussions

### 3.1. Shielding Effectiveness of Tempered Glass

Table 3, Table 4 and Table 5 indicate the results of SE test of each specimen depending on the frequency range. Table 3 indicates the results of tempered glass and metal plates. Table 4 indicates the results of ATMS coatings by Zn-Al depending on its thickness. Table 5 shows the results of ATMS coatings by Cu depending on its thickness.

In case of tempered glass, it was found that the average SE was 0.9 dB due to more brittleness than metallic and composite materials. Therefore, the tempered glass exhibits lower SE values than others. Based on this result, it can be seen that the tempered glass had little influence on the SE of the ATMS coating.

### 3.2. Shielding Effectiveness of Metal Plate

**Figure 3:**
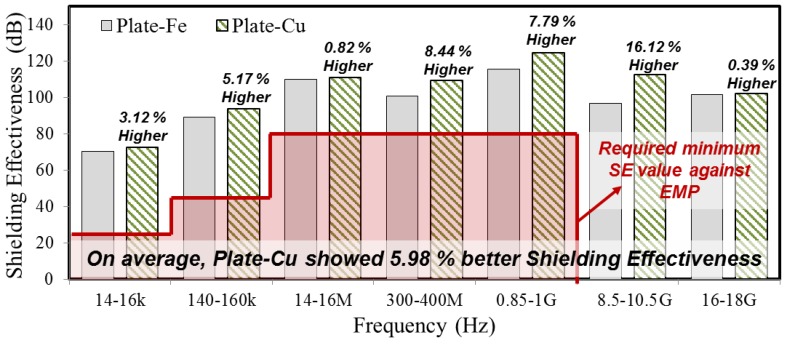
SE test results between Plate-Fe and Plate-Cu.

For the sake of clarity, SE values are explained in Figure 3, which represents the test results of Plate-Fe and Plate-Cu. It was found that both Plate-Fe and Plate-Cu satisfied the minimum SE throughout the entire frequency range. Plate-Fe showed and average of 97.7 dB SE and Plate-Cu showed average 103.6 dB SE (Table 3). Based on the comparison of the SE with regards to metal type, Cu showed about 5.98% better SE than Fe on average throughout all studied frequency ranges (Figure 3). Thus, as an EMP shielding material, Cu showed better SE than Fe and this might be due to the conductivity and mechanical properties, which affects the SE values [40]. The required SE values against each studied frequency are shown by a red line in Figure 3.

### 3.3. Shielding Effectiveness of ATMS Coating

**Figure 4:**
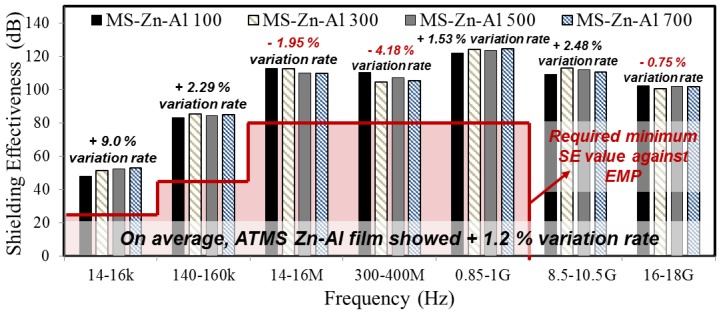
SE test result of ATMS coatings by Zn-Al in regards to thickness.

Figure 4 indicates the SE test results of MS-Zn-Al specimens with different coating thicknesses. It was found that all specimens satisfied the required minimum SE value, regardless of the coating thickness. Even with only 100 μm thickness, the minimum thickness which ATMS coating is applied, it showed an average of 9% higher SE than the required minimum SE values. The test results, with regards to coating thickness, showed a tendency that there was an increase of SE with an increase of thickness (14–16 kHz, 140–160 kHz, 0.85–1 GHz, and 8.5–10.5 GHz range). On the other hand, there was also a decrease in SE with an increase of thickness (14–16 MHz, 300–400 MHz, and 16–18 GHz range). This means that thickness is not necessarily relevant to the improvement of SE, in the range of μm thickness.

**Figure 5:**
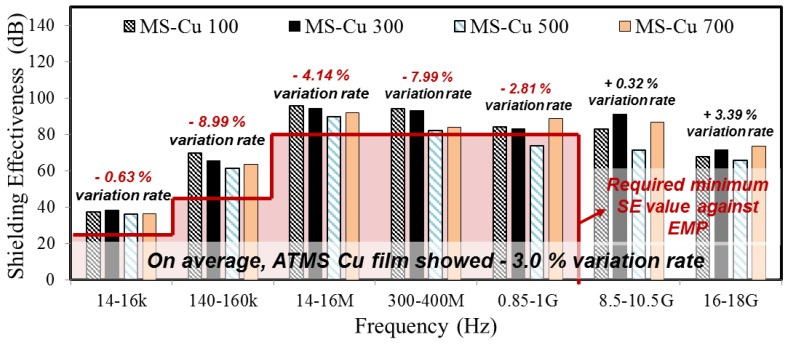
SE test result of ATMS coatings by Cu in regards to thickness.

The symbol on bars of Figure 5, i.e., (+) represents the increase of shielding rate while (−) represents the decrease of shielding rate on average variation rate (%) of Zn-Al specimens. As a result, it showed that the maximum increase rate on average was about +9% (14–16 KHz) and the decrease rate on average was about −4.18% (300–400 MHz). On average, the variation rate throughout entire frequency range showed about +1.2% increase. It can be analyzed that ATMSM formed a stable Zn-Al coating that is able to shield any electromagnetic wave properly. Thus, it is judged that the thickness of ATMS coating by Zn-Al does not cause a big difference, as EMP shielding material. It showed consistent SE value regardless of thickness.

**Figure 6:**
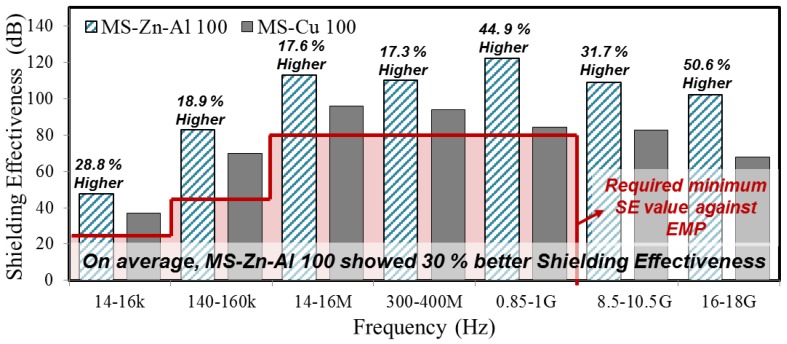
SE test result between MS-Zn-Al 100 and MS-Cu 100.

Figure 6 shows the SE test results of MS-Cu. It was found that the ATMS coatings by Cu also satisfied the required minimum SE value, regardless of the thickness. However, differently from the test result by Zn-Al coating, there is, mainly, a decrease of SE with an increase in thickness. In addition, the variation rate fluctuated more than that of the Zn-Al coating. It seems that ATMSM forms a relatively unstable Cu coating. This can provide an unsatisfying SE value according to the standards.

The MS-Zn-Al 100 and MS-Cu 100 specimens performed better than other thicknesses of coatings; therefore, the SE test results of these two coatings are shown in Figure 6. As a result, MS-Zn-Al 100 showed higher SE than MS-Cu 100 from minimum +17.3% (300–400 MHz range) to maximum +44.9% (0.85–1 GHz). This result indicates that ATMS coatings by Zn-Al have about 30% higher SE, on average, than the ATMS-Cu coating in terms of the entire frequency range. It is judged that this is because the density of the Zn-Al coating was more compact than that of the Cu.

**Figure 7:**
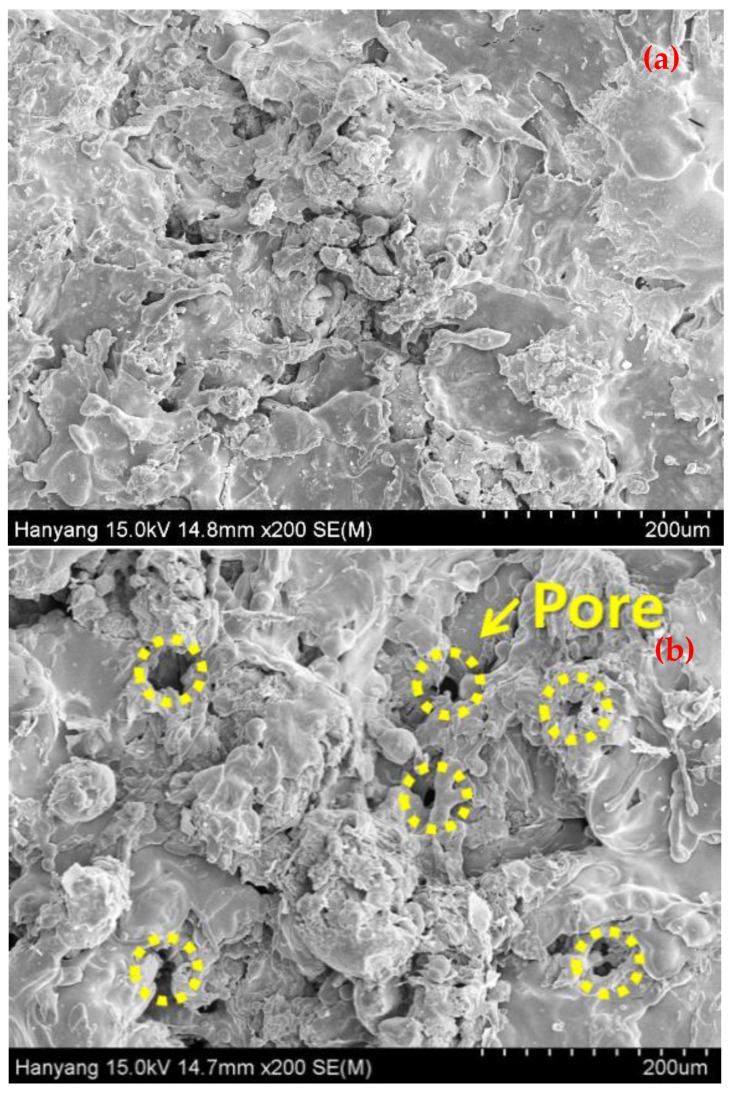
SEM image of the specimen surface of (**a**) MS-Zn-Al 100 and (**b**) MS-Cu 100.

To corroborate the above findings, scanning electron microscopy (SEM, Philips XL 30, North Billerica, MA, USA) results of MS-Zn-Al 100 and MS-Cu 100 are shown in Figure 7. When the ATMS method is applied on the substrate, the metal particles form a coating in a gelatinous state, which is between a liquid and solid state. In this process, a metal that has a lower melting temperature easily forms this gelatinous state and the ATMS coating accumulates more densely [34]. The melting temperatures of Zn, Cu, and Al, are 419.53 °C, 1084.62 °C, and 660.32 °C, respectively. The surface of MS-Zn-Al 100 in Figure 7a was formed with lesser pores and more densely than that of MS-Cu 100 in Figure 7b. When applying Cu as ATMS coating, it was shown that Cu did not form a dense coating on the substrate rather it forms more pores/cracks on top surface evident from Figure 7b. In addition, it created many pores between metal particles. The reason is that Cu was not easy to change its particle in a gelatinous state than Zn or Al due to its higher melting point. This result indicates that there is more possibility for electromagnetic wave to penetrate the Cu coating through the connected pores in actual EMP occurrence. When the principle on shielding effectiveness by metallic material was explained, it was mentioned that a metal layer with no hole or crack can improve SE against electromagnetic wave. From the analysis, SE test result shown in Figure 5 can be explained why ATMS coating by Cu showed unstable SE value. In spite of increased coating thickness, due to the formation of many pores in the coating, it could not show consistent SE against electromagnetic waves. Thus, it is judged that when applying the ATMS method as the EMP shielding material, Zn-Al can better SE than Cu.

### 3.4. Shielding Effectiveness between Metal Plate and ATMS Coating

**Figure 8:**
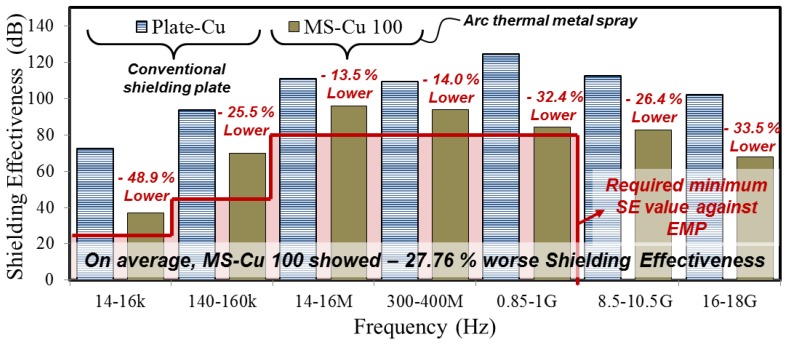
SE test result between metal plate and ATMS coating by Cu.

Figure 8 indicates the SE test results of metal plate (plate-Cu) and ATMS coating by Cu (MS-Cu 100). It shows that MS-Cu 100 has a lower SE than plate-Cu, from a minimum of −13.54% (14–16 MHz) to a maximum of −48.9% (14–16 kHz) and, on average, −27.76% for the entire frequency range. From the results in Figure 4 and Figure 5, it was found that the SE of metallic materials was not largely influenced by the coating thickness. Thus, it is judged that the lower SE value of MS-Cu 100 than plate-Cu is due to the large number of pores in MS-Cu 100. Based on this result, it is thought that Cu is not a suitable replaceable material for the metal plate when applying the ATMS method.

**Figure 9:**
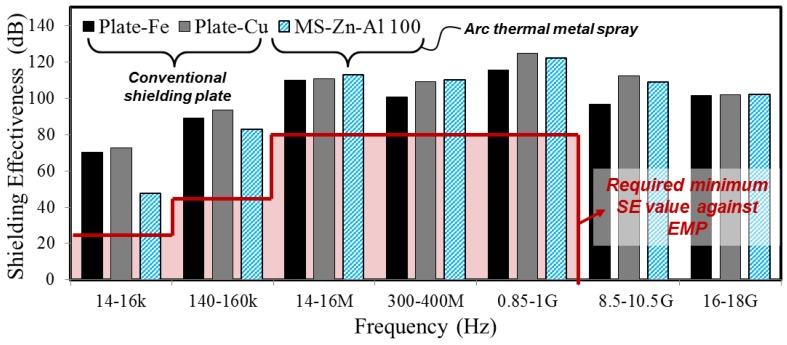
SE test result between metal plate and ATMS coating by Zn-Al.

Figure 9 indicates SE test results of Plate-Fe and Plate-Cu, which are metal plates, and ATMS coating by Zn-Al (MS-Zn-Al 100). As a comparison of SE between MS-Zn-Al 100 and metal plates, MS-Zn-Al 100 showed a lower SE in the low-frequency range (14–16 kHz, 140–160 kHz) than metal plates. However, in the other frequency ranges (14–16 MHz, 300–400 MHz, 0.85–1 GHz, 8.5–10.5 GHz, 16–18 GHz), MS-Zn-Al 100 showed similar or more improved SE than metal plates. Based on these results, it is judged that the ATMS method by Zn-Al has high possibility on actual application, instead of conventional EMP shielding plate.

## 4. Conclusions

This study evaluated the shielding effectiveness against EMP of the ATMS method in order to investigate the applicability of ATMS coating as a replacement material for conventional EMP shielding plate. As a SE test result, ATMS coating by Zn-Al satisfied the required SE according to the standards. In addition, it showed similar shielding performance in comparison with metal plate. Therefore, it is judged that ATMS coating has a high probability of actual application as a new EMP shielding material.

## Figures and Tables

**Table 1:** Experimental variables for the evaluation of the shielding effectiveness (SE) test against EMP.

Experimental Variables	Experimental Parameters
EMP Shielding method	Conventional shielding plate
	Arc thermal metal spray
EMP Shielding material	Fe
	Cu
	Zn-Al
Thickness of ATMS coating	100 μm
	300 μm
	500 μm
	700 μm

**Table 2:** List of specimens for the SE test.

No.	Specimens	Shielding Material	Shielding Method	Coating Thickness (μm)
1	Tempered glass^1^	-	-	-
2	Plate^2^-Fe	Fe (steel)	Metal plate	3000
3	Plate-Cu	Cu (copper)		3000
4	MS^3^-Zn-Al 100	Zn-Al^4^ (zinc and aluminum)	Arc thermal metal spraying method	100
5	MS-Zn-Al 300			300
6	MS-Zn-Al 500			500
7	MS-Zn-Al 700			700
8	MS-Cu 100	Cu		100
9	MS-Cu 300			300
10	MS-Cu 500			500
11	MS-Cu 700			700

Tempered glass^1^: Substrate for application of ATMS method; Plate^2^: Conventional EMP shielding plate (metal plate); MS^3^: Specimens which Arc thermal metal spray is applied; Zn-Al^4^: Zn and Al wire is applied at the same volume ratio (50:50), respectively.

**Table 3:** SE test results of specimens 1–3.

Frequency (Hz)	Required Minimum SE (dB)	Shielding Effectiveness (dB)		
		Tempered Glass	Plate-Fe	Plate-Cu
14–16 k	23.5	0.4	70.4	72.6
140–160 k	43.5	0.1	89.0	93.6
14–16 M	80	1.5	109.9	110.8
300–400 M	80	0.5	100.7	109.2
0.85–1 G	80	1.5	115.6	124.6
8.5–10.5 G	-	1.3	96.8	112.4
16–18 G	-	1.1	101.5	101.9
Average SE (dB)		0.91	97.70	103.59

**Table 4:** SE test results of specimens 4–7.

Frequency (Hz)	Required Minimum SE (dB)	Shielding Effectiveness (dB)			
		MS-Zn-Al 100	MS-Zn-Al 300	MS-Zn-Al 500	MS-Zn-Al 700
14–16 k	23.5	47.8	51.2	52.3	52.8
140–160 k	43.5	82.9	85.4	84.2	84.8
14–16 M	80	112.7	112.3	109.7	109.5
300–400 M	80	110.1	104.4	107.0	105.1
0.85–1 G	80	122.0	123.9	123.4	124.3
8.5–10.5 G	-	108.9	112.8	111.7	110.3
16–18 G	-	102.1	100.5	102.0	101.5
Average SE (dB)		98.07	98.64	98.61	98.33

**Table 5:** SE test results of specimens 8–11.

Frequency (Hz)	Required Minimum SE (dB)	Shielding Effectiveness (dB)			
		MS-Cu 100	MS-Cu 300	MS-Cu 500	MS-Cu 700
14–16 k	23.5	37.1	38.2	36.0	36.4
140–160 k	43.5	69.7	65.4	61.4	63.5
14–16 M	80	95.8	94.1	89.6	91.8
300–400 M	80	93.9	93.2	82.1	83.9
0.85–1 G	80	84.2	83.0	73.7	88.8
8.5–10.5 G	-	82.7	91.2	71.2	86.5
16–18 G	-	67.8	71.3	65.5	73.5
Average SE (dB)		75.89	76.63	68.50	74.91

## Supplementary Materials

The following are available online at http://www.mdpi.com/1996-1944/10/10/1155/s1, Figure S1: Shielding principle of metal material against electromagnetic wave, Figure S2: Coating thickness and error bar of Zn-Al and Cu coating, Figure S3: The required shielding effectiveness in terms of EMP shielding room, Figure S4: Schematic diagram of SE test for specimens, Figure S5: Cross-sectional diagram of SE test for installation of specimens, Table S1: Frequency range of SE test.
