# Supplementary material for: Study on the Shielding Effectiveness of an Arc Thermal Metal Spraying Method against an Electromagnetic Pulse
Source: Materials (Basel). 2017 Oct 4;10(10):1155. doi: 10.3390/ma10101155 (PMC5666961; doi:10.3390/ma10101155)
Supplement: Supplementary file 1 [file materials-10-01155-s001.docx]

Supplementary Materials: Study on the Shielding Effectiveness of an Arc Thermal Metal Spraying Method against an Electromagnetic Pulse

Han-Seung Lee, Hong-Bok Choe, In-Young Baek, Jitendra Kumar Singh and
Mohamed A. Ismail

**
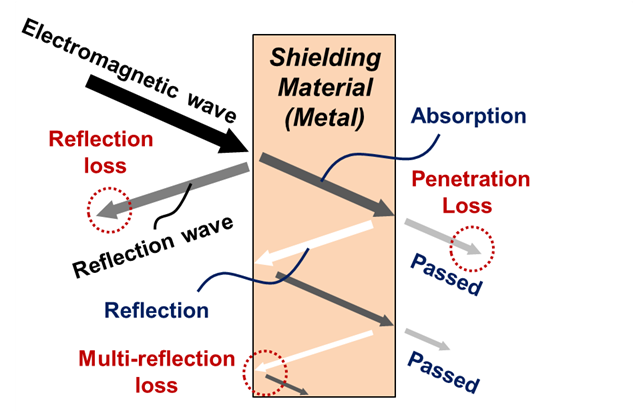
**

**Figure S1:** Shielding principle of metal material against electromagnetic wave.

**Figure S2:** Coating thickness and error bar of Zn-Al and Cu coating


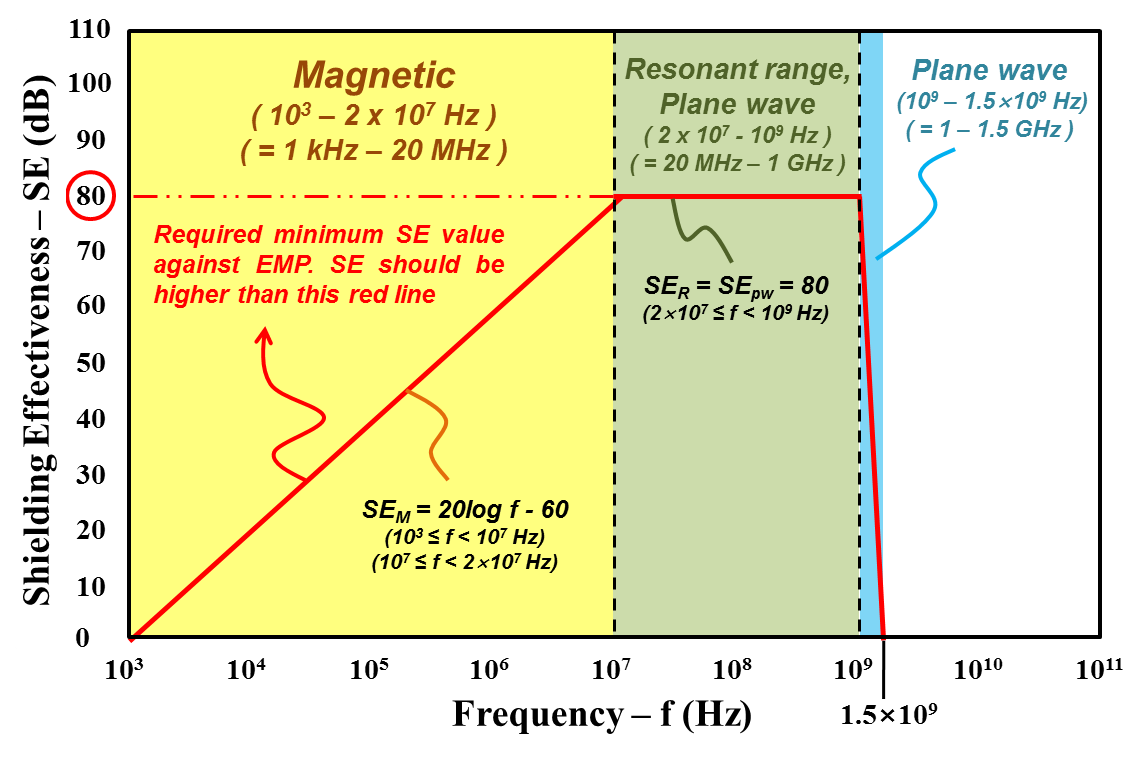


**Figure S3:** The required shielding effectiveness in terms of EMP shielding room.


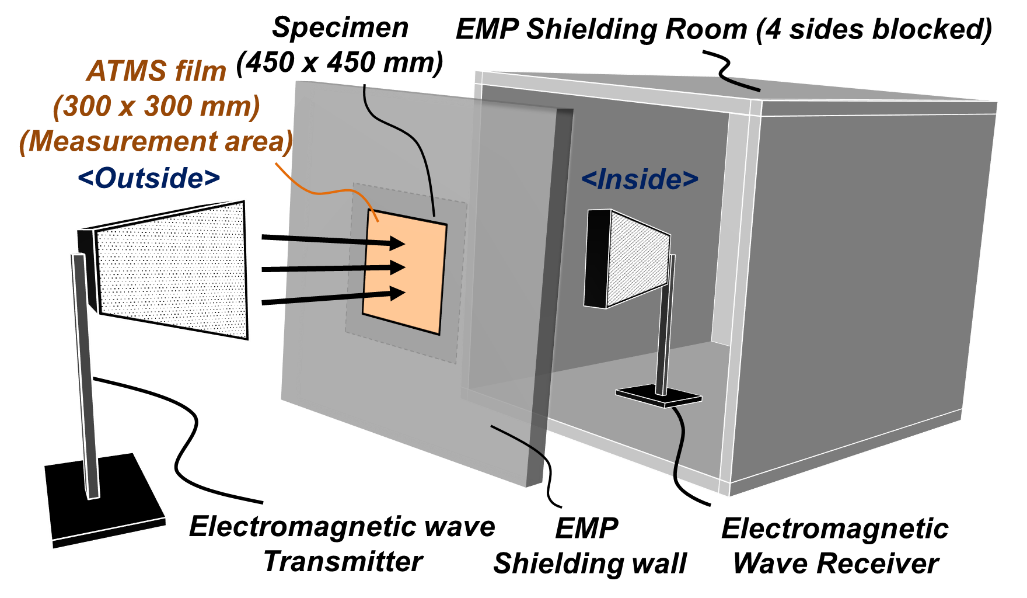


**Figure S4:** Schematic diagram of SE test for specimens.


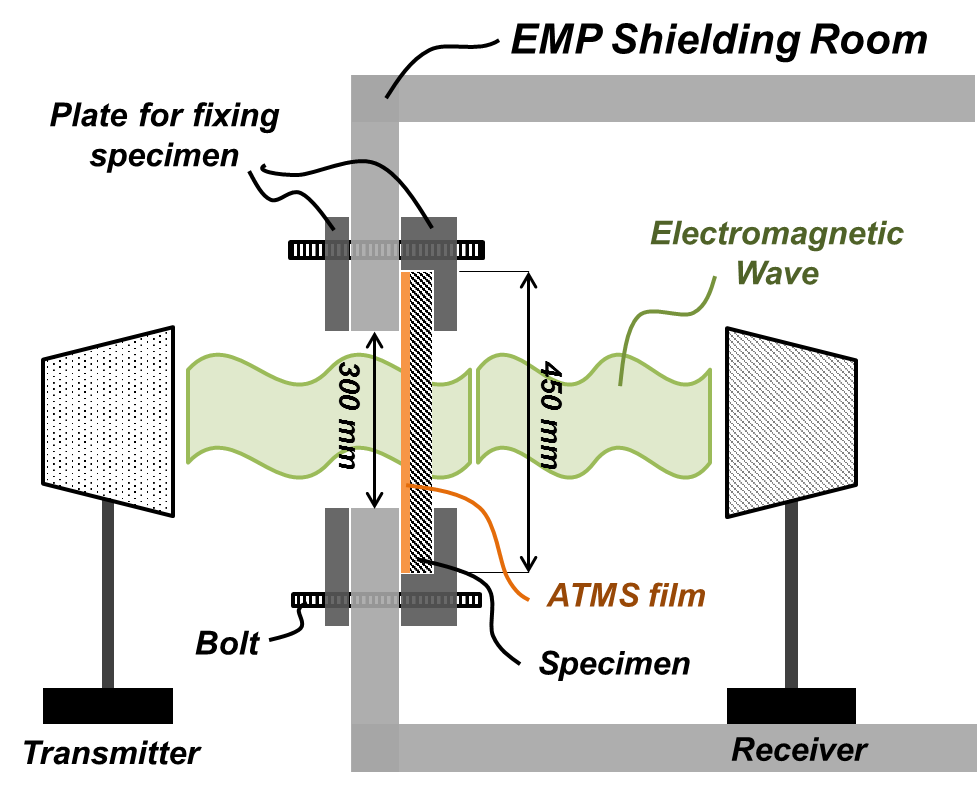


**Figure S5:** Cross-sectional diagram of SE test for installation of specimens.

**Table S1:** Frequency range of SE test.

| No. | Test Frequency Range (Hz) | Required Minimum SE (dB) | Applied Field | Type of Electromagnetic Wave |
| --- | --- | --- | --- | --- |
| 1 | 14–16 k | Above 23.5 dB | Private and Military facility (EMP) | Magnetic  (10^3^–2 × 10^7^ Hz) |
| 2 | 140–160 k | Above 43.5 dB |  |  |
| 3 | 14–16 M | Above 80 dB |  |  |
| 4 | 300–400 M |  |  | Resonant range and Plane wave (2 × 10^7^–10^9^ Hz) |
| 5 | 0.85–1 G |  |  |  |
| 6 | 8.5–10.5 G | - | Private facility (EMC^1^) | Plane wave  (10^9^–1.5 × 10^9^ Hz) |
| 7 | 16–18 G | - |  |  |
| Index | EMC^1^: Electromagnetic Compatibility | | | |

© 2017 by the authors. Submitted for possible open access publication under the
terms and conditions of the Creative Commons Attribution (CC BY) license (http://creativecommons.org/licenses/by/4.0/).
